# Evaluation of Cottonseed Meal as an Alternative to Fish Meal in Diet for Juvenile Asian Red-Tailed Catfish *Hemibagrus wyckioides*

**DOI:** 10.1155/2023/1741724

**Published:** 2023-01-06

**Authors:** Biwei Li, Linhai Su, Yan Sun, Han Huang, Junming Deng, Zhiyong Cao

**Affiliations:** ^1^College of Animal Science and Technology, Yunnan Agricultural University, Kunming 650201, China; ^2^College of Fisheries, Guangdong Ocean University, Zhanjiang 524088, China; ^3^College of Big Data, Yunnan Agricultural University, Kunming 650201, China

## Abstract

A 10-week trial was performed to investigate the effects of replacing fishmeal with cottonseed meal (CSM) on the growth rate, protein metabolism, and antioxidant response of Asian red-tailed catfish *Hemibagrus wyckioides*. Five isonitrogenous and isocaloric diets (C0, C8.5, C17.2, C25.7, and C34.4) were prepared to contain 0%, 8.5%, 17.2%, 25.7%, and 34.4% CSM replacing fishmeal, respectively. The weight gain, daily growth coefficient, pepsin, and intestinal amylase activities initially increased and then decreased with the raising dietary CSM levels; the highest values were observed in the C17.2 group (*P* < 0.05). However, feed cost exhibited the opposite trend. With the increasing dietary CSM levels, the protein efficiency ratio and intestinal trypsin activity decreased but feed conversion rate increased gradually; while no differences were observed among the C0, C8.5, and C17.2 groups (*P* > 0.05). Dietary CSM inclusion regardless of levels increased the plasma growth hormone level as well as hepatic aspartate aminotransferase (AST) and *γ*-glutamyl transpeptidase activities but decreased the plasma glutamate dehydrogenase and AST activities (*P* < 0.05). With the increasing dietary CSM levels, the plasma alkaline phosphatase (AKP) and hepatic superoxide dismutase activities decreased but malondialdehyde content increased gradually, while no differences were observed among the C0, C8.5, and C17.2 groups (*P* > 0.05). The plasma immunoglobulin M content and hepatic glutathione reductase activity initially increased but then decreased with the raising dietary CSM levels; the highest values were found in the C17.2 group. These results indicated that dietary CSM inclusion level up to 17.2% improved the growth rate, feed cost, digestive enzyme activity, and protein metabolism without compromising antioxidant capacity of *H. wyckioide*, whereas these parameters were depressed by further inclusion of CSM. CSM is a potentially cost-effective alternative plant protein source in diet of *H. wyckioide*.

## 1. Introduction

As the most important protein source in aquafeed, fishmeal (FM) possesses not only balance amino acids and active substances (e.g. taurine, hydroxyproline, cholesterol) but also incomparable palatability [1]. However, FM can no longer meet the needs of aquaculture because of decreasing fishery resources and increasing FM demand; and which become an enormous barrier for aquaculture expansion. Thus, the pursuit for alternatives to FM has always been hotspot of aquaculture [2]. Among alternative protein sources for FM, plant protein sources appear to be the most appropriate because of their availability, eco-friendly, sustainability, and price [3]. Cottonseed meal (CSM) is a by-product obtained from the process of extracting oil from cottonseed. According to the China Statistical Yearbook 2021, the planting area of cottonseed in China reached 3.03 million hectares, and the output reached 10.32 million tons. Behind soybean meal and rapeseed meal, CSM ranks third in terms of tonnage among the commercially available plant protein sources. CSM is considered a valuable plant protein source as an FM substitute in aquafeed because of its relatively favorable amino acid profiles [4]. Previous studies showed that CSM could replace 15–50% FM without compromising growth rate of Ussuri catfish *Pseudobagrus ussuriensis* [5], blunt snout bream *Megalobrama amblycephala* [6], grass carp *Ctenopharyngodon idellus* [7], silver sillago *Sillago sihama* [8], red drum *Sciaenops ocellatus* [9], and turbot *Scophthalmus maximus* [10, 11]. However, the further substitution were limited by several factors, including relatively low contents of lysine and methionine as well as high level of free gossypol [12].

Asian red-tailed catfish *Hemibagrus wyckioides*, an omnivorous freshwater fish, is widely distributed in the Mekong River Basin [13]. This species is very popular with farmers and consumers for its fast growth rate, large size, extensive adaptability, and superior resistance to disease [14, 15]. In addition, this catfish do not exhibit cannibalism phenomenon unlike other catfish [16]. Although this species is widely cultured in ponds and cages throughout the Lancang-Mekong River Area, there is no special compound feed for *H. wyckiodies* due to limited information on the requirements of nutrients [13, 17–19] and utilization of feed ingredients [20, 21]. Hence, an assessment of the utilization efficiency of various plant protein sources by *H. wyckioides* is urgently needed. To date, there is no data on the influence of CSM inclusion in diet for *H. wyckioides*. The aim of the present study was to assess the effects of replacing FM with CSM on growth performance, protein metabolism, and antioxidant response of *H. wyckioides*.

## 2. Materials and Methods

### 2.1. Experimental Diets

Using FM (crude protein, 73.6%) and CSM (crude protein, 49.6%; free gossypol, 1.12 mg/g) as the primary protein sources, five isonitrogenous (41% crude protein) and isocaloric (21 kJ/g gross energy) diets (C0, C8.5, C17.2, C25.7, and C34.4) were prepared to replace 0%, 12%, 24%, 36%, and 48% FM protein with 0%, 8.5%, 17.2%, 25.7%, and 34.4% CSM, respectively. DL-methionine, L-lysine, and L-leucine were also supplemented to each diet to provide equal amounts of methionine, lysine, and leucine in all experimental diets. The feed ingredients and proximate composition of experimental diets are listed in Table 1, and the corresponding amino acid profiles are shown in Table 2.

All feed ingredients except lipid sources were crushed and passed through a sieve with a diameter of 320 *μ*m. Soybean lecithin was preblended in the mixture of soybean oil and fish oil. All ingredients were thoroughly mixed; the lipid mixture was added, and then thoroughly mixed again. Suitable water was added to make a dough, and the dough was squeezed into 1.5-mm pellet by a pellet feed maker (KS-180; Jiangsu Jingu Rice Mill Co., Ltd., Zhenjiang, China). The wet pellets were dried at 40°C and then stored at −20°C until use.

### 2.2. Fish and Experimental Procedure

Before the initiation of feeding trial, juvenile *H. wyckioides* originated from the same batch were fed commercial rainbow trout diet (TR-2242, Chile) for 14 days to acclimate the experimental conditions. After 24-h fast, healthy and uniform juveniles (initial body weight 3.21 ± 0.02 g) were selected and randomly assigned into five dietary groups with triplicates of 35 juveniles per tank (1.0 m × 0.6 m × 0.5 m). During the feeding trial, the experimental diets were manually fed twice a day (07 : 00, 17 : 00) to apparent satiation for 10 weeks. The cultivation water was recirculated through a filtration system composed of filter sponge, coral stone and active carbon to remove particulate matter and hazardous substances, and water temperature was kept at 27 ± 1°C by a heating rod. All tanks were provided with continuous aeration (dissolved oxygen ≥6 mg/L) and natural photoperiod (13.5 h light/10.5 h dark at the beginning of July and 12 h light/12 h dark at the end of September).

### 2.3. Sample Collection

After the feeding trail, all fish were anesthetized with eugenol (1 : 12000; Shanghai Reagent Corporation, Shanghai, China) after 24-h fast. All fish from each tank were weighed and counted to calculate the growth rate and feed utilization. Five fish per tank were randomly collected and stored at –20°C for body composition analysis. Blood samples were collected from the caudal vein of ten fish per tank using a sterile 1-ml syringe. One part of blood sample was transferred into an Eppendorf tube without anticoagulant, and the other part was collected in a heparinized tube. Serum/plasma samples were allowed to clot for 4 h at 4°C, then centrifuged at 4000 g for 10 min at 4°C, the supernatants were collected and stored at –80°C. Liver, stomach, and midgut (the part from anterior valvula intestine to anterior rectum) were dissected from five fish per tank and stored at –80°C until analysis. Three additional samples of liver and dorsal muscle were collected from each tank and pooled in a 1.5-ml Sterile cryovials (Axygen®), and stored at –80°C for analysis of relative expression of protein metabolism-related genes (mammalian target of rapamycin (mTOR), adenosine monophosphate deaminase 1 (AMPD1), glutamate dehydrogenase (GDH), and insulin-like growth factor 1 (IGF-1).

### 2.4. Chemical Analysis

#### 2.4.1. Proximate Composition

The proximate composition of feed ingredients, experimental diets, and whole-body samples were measured following the method of AOAC [22]. Dry matter was determined by drying the sample to a constant weight at 105°C; crude protein content was examined by the Kjeldahl method (N × 6.25); crude lipid content was determined using the Soxhlet method with ether extraction; crude ash content was determined after burning in a muffle furnace at 550°C for 6 h. A bomb calorimeter (Parr 1351; Parr Instrument Co., Moline, IL, USA) was used to measure gross energy. Free gossypol content in diets was determined by high-performance liquid chromatography [23]. The amino acid composition of diets was determined using the method described by Mai et al. [24].

#### 2.4.2. Digestibility Enzyme Activity

To obtain an adequate crude enzyme extract solution, the amount of physiological saline solution (0.9% NaCl) needed to add to the wet stomach/midgut sample was determined by a preliminary study. Approximately 0.2 g wet stomach/midgut plus 1.8 mL cold 0.9% NaCl solution were transferred to a 5-mL centrifugal tube and then homogenized. The homogenate was centrifuged at 6,000 g for 15 min at 4°C (MX-160; Tomy Industry, Tokyo, Japan). The supernatant was transferred to a new test tube for analysis of digestive enzyme activity. Pepsin, trypsin, lipase, and amylase activities were assayed by commercial kits (Nanjing Jiancheng Bioengineering Institute, Nanjing, China). One unit of pepsin/trypsin activity was defined as 1 mmol of tyrosine equivalent released per min at 37°C. One unit of lipase activity was defined as the amount of enzyme required to produce 1 *μ*mol of p-nitrophenol at 37°C for 1 minute. One unit of amylase activity was defined as the amount of enzyme hydrolyzing 0.1 mg of starch in 30 minutes at 37°C. Specific enzyme activity was expressed as enzyme activity per gram protein. The amount of soluble protein in supernatant was determined following the approach of Marion and Bradford [25].

#### 2.4.3. Protein Metabolism-Related Parameters

Blood urea nitrogen (BUN) and albumin contents as well as alanine aminotransferase (ALT) and aspartate aminotransferase (AST) activities in plasma were measured using commercial kits (Nanjing Jiancheng Bioengineering Institute, Nanjing, China). Plasma insulin, growth hormone (GH) and IGF-1 levels, and GDH and AMPD activities were measured by enzyme-linked immunosorbent assay (ELISA) kits (R&D Systems Inc., Minneapolis, MN). Hepatic GDH, AMPD, AST, and ALT activities were determined using the same kits as for plasma and expressed as enzyme activity per gram protein. Hepatic *γ*-glutamyl transferase (*γ*-GT) activity was measured by ELISA kit (R&D Systems Inc., Minneapolis, MN) and expressed as enzyme activity per gram protein.

#### 2.4.4. Antioxidant-Related Parameters

Immunoglobulin M (IgM) and malondialdehyde (MDA) contents, alkaline phosphatase (AKP) activity in plasma; and superoxide dismutase (SOD), catalase (CAT), peroxidase (POD), glutathione peroxidase (GSH-Px), glutathione reductase (GR), AKP, and lactate dehydrogenase (LDH) activities in liver were analyzed using the commercial kits (Nanjing Jiancheng Bioengineering Institute, Nanjing, China). Nitric oxide (NO) content in liver was measured using a nitrate reductase method [26].

#### 2.4.5. RNA Extraction, cDNA Synthesis, and Real-Time PCR Analysis

The procedures of RNA isolation, reverse transcription, and quantitative real-time PCR were performed as described by Zhang et al. [20]. Briefly, total tissue RNA was extracted with an RNAiso Plus Kit (Takara Bio, Inc., Dalian, China) and then quantified by a Nanodrop 2000 spectrophotometer (Thermo Scientific, Shanghai, China). Agarose gel (1%) electrophoresis was used to confirm the completeness of RNA sequences and then total RNA was immediately reverse-transcribed to obtain cDNA (Thermo Scientific Revert Aid Firs Strand cDNA Synthesis Kit), which was stored at –20°C until use. The mRNA levels of target genes were assessed using real-time PCR. Primers of target genes were devised by using Primer Premier 5.0 software in accordance with the nucleotide sequences of similar species in GenBank. Gene sequences that could not be acquired in GenBank were cloned, amplified, and sequenced. The new gene sequences were used to design specific real-time PCR primers. All primers were synthesized by Sangon Biotech Co., Ltd. (Shanghai, China). The template amount was normalized by using the *β*-actin gene as an internal reference (Table 3). The 2^–*ΔΔ*Ct^ method was used to analyze the relative expression level of the above genes [27].

#### 2.4.6. Calculation and Statistical Analysis

The following formulas were used to calculate various parameters and indices:

(1) $$\begin{array}{c} \text{Mean metabolic body weight }\left(\text{MBW}\right)=\frac{\left[{\left(Wi/1000\right)}^{0.75}+{\left(W_{f}/1000\right)}^{0.75}\right]}{2},\\ \text{Feed intake }\left(\text{g}/\text{kg MBW per day}\right)=\frac{\text{DI}/\text{MBW}}{d},\\ \text{Weight gain }\left(\text{WG}\right)=\frac{\left(W_{f}–W_{i}\right)}{Wi},\\ \text{Daily growth coefficient }\left(\text{DGC},\%/\text{d}\right)=\frac{{W_{f}}^{1/3}–{W_{i}}^{1/3}}{d}\times 100,\\ \text{Feed conversion rate }\left(\text{FCR}\right)=\frac{\text{DI}}{W_{f}–W_{i}},\\ \text{Protein efficiency ratio }\left(\text{PER}\right)=\frac{W_{f}–W_{i}}{\text{protein intake}},\\ \text{Feed cost}\left[\text{US\$}/\left(\text{kg fish gain}\right)\right]=\text{FP}\times \text{FCR}. \end{array}$$

where *W*_*i*_ and W_f_ are the initial and final body weights (g); *d* is the feeding days; and DI is the dry diet intake per fish (g DM/fish); and FP (feed price, US$/kg feed) is calculated as feed ingredients cost + manufacture cost.

All data were analyzed using one-way analysis of variance (ANOVA) in SPSS 17 (SPSS Inc., Chicago, IL, USA) for Windows. If the difference was significant (*P* < 0.05), Tukey's test was used for multiple comparisons. All results are expressed as mean ± standard error (*n* = 3). Regression analysis was also performed to determine the degree of relationship between various parameters and replacement level of FM by CSM.

## 3. Results

### 3.1. Growth Performance

After the feeding trial, the survival rate of fish ranged from 96.67% to 98.89%, no statistical difference was found among the dietary groups (*P* > 0.05; Table 4). The final body weight (*y* = 20.240 + 14.352*x*–31.630*x*^2^, *P* = 0.278), WG (*y* = 5.353 + 4.512*x*–9.673*x*^2^, *P* = 0.318), and DGC (*y* = 2.240 + 1.092*x*–2.397*x*2, *P* = 0.313) initially increased and then declined with the raising dietary CSM content; the highest growth rate was observed in the C17.2 group. Conversely, the feed cost firstly decreased and then increased slightly with the raising dietary CSM content; the lowest value was found in the C17.2 group (*y* = 1.251–0.382*x* + 0.493*x*^2^, *P* = 0.005). The PER (*y* = 2.747–0.414*x*, *P* = 0.002) decreased but FCR (*y* = 0.885 + 0.133*x*, *P* = 0.006) increased with the raising dietary CSM content, but no significant differences were found among the C0, C8.5, and C17.2 groups. However, no statistical difference was found in the feed intake (g/kg MBW per day) among the dietary groups (*P* > 0.05).

### 3.2. Digestive Enzyme Activity

The activities of pepsin (*y* = 15.430 + 23.471*x*–50.761*x*^2^, *P* = 0.157) and intestinal amylase (*y* = 2.792 + 2.198 *x*–6.101*x*^2^, *P* = 0.334) initially raised and then decreased with the increasing dietary CSM content (Table 5); the highest activities were observed in the C17.2 group. The intestinal trypsin activity (*y* = 1.309–0.683*x*, *P* = 0.007) decreased gradually with the raising dietary CSM level, and that was significantly higher in the C0 group compared to the C25.7 and C34.4 groups (*P* < 0.05). However, no statistical difference was found in the intestinal lipase activity among the dietary groups (*P* > 0.05).

### 3.3. Protein Metabolism-Related Parameters

The plasma GH content (*y* = 17.758 + 56.866*x*–103.737*x*^2^, *P* < 0.001) and AMPD activity (*y* = 14.234 + 57.976*x*–97.520*x*^2^, *P* = 0.019) as well as hepatic AST activity (*y* = 13.362 + 111.039*x*–204.613*x*^2^, *P* = 0.005) initially increased and then decreased with the increasing dietary CSM content; and these values were lower in the C0 group compared to the other groups (*P* < 0.05, Table 6). The plasma GDH content in the C0 group or hepatic AMPD activity in the C8.5 group were higher than that in the other groups; in contrast, the hepatic *γ*-GT activity was lower in the C0 group as compared to the other groups (*P* < 0.05). The plasma AST activity (*y* = 29.249–44.236*x*, *P* < 0.001) gradually declined with the raising dietary CSM content. However, no statistical differences were found in plasma albumin, BUN, insulin, IGF-1 contents, and ALT activity as well as hepatic GDH and ALT activities among the dietary groups (*P* > 0.05).

The hepatic IGF-1 expression level was higher in the C0 group compared to the C8.5 and C17.2 groups (*P* < 0.05, Table 7). Conversely, the hepatic mTOR expression level was higher in the C8.5 group compared to the C0, C25.7, and C34.4 groups (*P* < 0.05). The relative expression levels of hepatic AMPD and muscle GDH were higher in the C0 group compared to the other groups (*P* < 0.05). The hepatic GDH expression level was higher in the C0, C17.2, and C34.4 groups compared to the C8.5 and C25.7 groups (*P* < 0.05). The muscle AMPD expression level was lower in the C8.5 group compared to the C17.2 and C34.4 groups (*P* < 0.05).

### 3.4. Immune and Antioxidant-Related Indexes

The plasma IgM content (*y* = 0.061 + 0.743*x*–1.455*x*^2^, *P* = 0.002) and hepatic GR activity (*y* = 8.452 + 14.149*x*–37.782*x*^2^, *P* = 0.049) initially increased and then decreased with the increasing dietary CSM content; and the highest values were found in the C17.2 group (*P* < 0.05; Table 8). The plasma MDA content (*y* = 4.497 + 28.814*x*, *P* < 0.001) increased gradually with the raising dietary CSM content, whereas no statistical difference was observed among the C0, C8.5, C17.2, and C25.7 groups (*P* > 0.05). Conversely, the plasma AKP (*y* = 16.747–31.908*x*, *P* < 0.001) and hepatic SOD activities (*y* = 33.946–9.580*x*, *P* = 0.047) declined generally with the raising dietary CSM content. The hepatic NO content was higher in the C0 group compared to the C8.5 group (*P* < 0.05). However, no statistical differences were observed in the hepatic CAT, POD, GSH-Px, AKP, and LDH activities among the dietary groups (*P* > 0.05).

### 3.5. Whole-Body Composition

The whole-body crude lipid content (*y* = 10.746–2.125*x*, *P* = 0.004) declined generally with the increasing dietary CSM content; and that was lower in the C34.4 group compared to the C0 and C17.2 groups (*P* < 0.05, Table 9). Conversely, the whole-body moisture content (y = 69.971 + 2.244*x*, *P* = 0.035) increased gradually with the raising dietary CSM content, and that was higher in the C34.4 group compared to the C0 group (*P* < 0.05). However, no statistical differences in the whole-body crude protein and ash contents were observed among the dietary groups (*P* > 0.05).

## 4. Discussion

CSM is a potentially cost-effective alternative plant protein source for fish, but the inclusion level of CSM in aquafeeds was limited due to the potential toxic effect of free gossypol and the deficient in essential amino acids (EAAs) such as lysine, methionine, and leucine [7, 28]. However, CSM is rich in arginine and phenylalanine [7]. In this study, lysine, methionine, and leucine were supplemented to the diets containing CSM to attain concentrations like the control diet (Table 2); arginine concentration in the experimental diets increased from 2.16% (Diet C0) to 3.21% (Diet C34.4) with the increase of dietary CSM level. Except for the above-mentioned amino acids, other EAAs were similar among the experimental diets. Additionally, free gossypol content in the diets raised from 0 g/kg (Diet C0) to 0.39 g/kg (Diet C34.4), the highest value exceeded the tolerance limit of tilapia *Tilapia aurea* (0.18 g/kg) [29], rainbow trout *Oncorhynchus mykiss* (0.25–0.29 g/kg) [30, 31], and channel catfish *Ictalurus punctatus* (0.30 g/kg) [32], but less than that of crucian carp *Carassius auratus gibelio*♀ × *Cyprinus carpio*♂ (0.64 g/kg) [33].

The present study indicated that the growth rate of *H. wyckioides* improved with the increasing dietary CSM content up to 17.2% and then decreased; 25.7% CSM could be incorporated into the diet without causing growth depression, but the inclusion level of 34.4% CSM depressed the growth of *H. wyckioides*. Similar results were reported in previous studies with grass carp [7], turbot [10], tilapia [34], and southern flounder *Paralichthys lethostigma* [35], the inclusion of suitable CSM replacing FM markedly improved the growth performance and feed utilization. Furthermore, FM can be completely replaced by CSM in diet of snubnose pompano *Trachinotus blochii* without adverse effect on growth, metabolism, and general health [12]. However, Wang et al. [9] suggested that dietary CSM inclusion linearly depressed the growth rate of red drum *Sciaenops ocellatus*. These discordant findings may be explained by the differences in fish species and size, CSM quality, diet composition, and culture conditions. The amount of CSM that can be incorporated in aquafeeds depends mainly on the balance of amino acid and the tolerance of fish to free gossypol [36]. In this study, the supplements of methionine, lysine, and leucine were added as needed to ensure that each diet contained the same concentrations of limiting amino acids. However, dietary arginine concentration gradually increased from 2.16% to 3.21% with the raising inclusion level of CSM. Thus, the growth promoting effect of 17.2% CSM (2.69% arginine) in diet was directly related to the improvement of dietary arginine level in this study. Additionally, the inclusion level of CSM was also determined by the tolerance of *H. wyckioides* to free gossypol. The present study found no growth depression in fish fed diets containing free gossypol levels ranging from 0.10 to 0.29 mg/g; however, the growth rate was depressed when free gossypol concentrations reached 0.39 mg/g. Thus, the current study demonstrate that 0.29 mg/g free gossypol is the tolerance threshold for *H. wyckioides*, the value is similar to that for rainbow trout (0.29 g/kg) [31] and channel catfish (0.30 g/kg) [32].

Additionally, feed cost is the main indicator reflecting economic benefit of FM replacement. In this study, the feed cost initially decreased and then increased with the raising dietary CSM levels with the lowest value in the C17.2 group, which indicating that feeding diets containing 17.2% CSM apparently reduced the feed cost and FM reliance of *H. wyckioides* farming.

As is well known, the digestibility and utilization of nutrients mainly depend on digestive enzyme activity [37]. Specifically, the hydrolysis of protein firstly depends on pepsin, and the subsequent digestive process is carried out by trypsin and chymotrypsin in intestine [38]. Lipase can be detected throughout the intestine but is most active in midgut [20]. Omnivorous fish can utilize carbohydrate more efficiently than carnivorous fish because of their higher intestinal amylase activity [39]. In this study, the activities of pepsin and amylase increased with the raising dietary CSM concentrations up to 17.2% but then decreased at higher CSM levels; trypsin activity did not decrease significantly until dietary CSM content reached up 25.7%. These findings indicated that the suitable dietary arginine concentrations (2.69%) induced by dietary CSM inclusion enhanced the digestive capacity of *H. wyckioides*, but further inclusion of dietary CSM depressed the activities of digestive enzymes. The reduced digestive enzymes activities in fish fed high levels of CSM in this study might be attributable to the high content of free gossypol in high CSM-based diets. Previous studies reported that the free gossypol from CSM may directly inhibit certain enzymes (e.g., pepsinogen, pepsin, and trypsin) by binding the free epsilon amino groups of lysine in the gastrointestinal tract, and thereby depressed the intestinal digestive enzymes activities [7, 40].

Protein metabolism is a dynamic process involving the balance between the synthesis and degradation of protein [41]. The mTOR is a serine/threonine kinase, which regulates the protein synthesis [19]. As upstream molecules, insulin/IGF-1 regulate the TOR signaling pathway and participate in a series of physiological processes including cell growth and proliferation [42]. Additionally, GH can activate the TOR signaling pathway, and thereby accelerate protein synthesis [43]. In this study, the plasma GH level and relative expression of hepatic mTOR firstly enhanced and then declined with the increasing dietary CSM content, consistent with the trend of growth performance. These results indicated that the suitable dietary CSM inclusion (8.5%–17.2%) might enhance the protein synthesis and thereby improved the growth performance of *H. wyckioides*, but those were restrained by the inclusion of 25.7% or more. The promoting effect of low CSM inclusion on protein synthesis may be related to the improvement of amino acids balance (increased arginine content) in diets, while the depressed protein synthesis might be explained as an effect of excessive free gossypol in diets with higher CSM concentrations. A similar observation has been found in blunt snout bream [44], turbot [45], and crab *Portunus trituberculatus* [4] fed diets with moderate CSM inclusion.

It is well known that the activities and relative expression levels of GDH, MAPD, *γ*-GT, AST, and ALT are correlated with the protein or amino acid catabolism [21]. As an essential enzyme in glutamate metabolism, GDH reversibly catalyzes deamination of glutamate with the production of ammonia [46]. AMPD is an indispensable contributor to ammonia production through its catalysis of the irreversible hydrolysis of AMP to IMP and NH_4_^+^ [20]. It is well known that ALT and AST can catabolize amino acids and transfer amino groups to *α*-keto acid [47]; *γ*-GT is linked to amino acid transport via the *γ*-glutamyl cycle [48]. In this study, dietary CSM inclusion regardless of level depressed the plasma GDH and AST activities as well as relative expression of AMPD in liver, GDH in liver and muscle, but increased the hepatic *γ*-GT and AST activities. The response discrepancies of these parameters in different tissues have been observed in several earlier studies with Ussuri catfish [5] and turbot [10] fed diets with various contents of CSM. These results indicated that dietary CSM inclusions altered the amino acid catabolism in *H*. *wyckioides*, which may be related to the synchronous influences of dietary arginine and free gossypol contents. The improvement of amino acids balance (increased arginine content) in diets might inhibit the protein degradation, while free gossypol had certain negative effect on protein metabolism. Free gossypol is easy to combine with lysine, resulting in the decrease of lysine activity [4]. Thus, the high level of arginine present in CSM can promote protein synthesis but inhibit protein degradation as energy source, and thereby improved the growth performance, whereas the high concentration of free gossypol may disturb the protein metabolism of *H*. *wyckioides*.

As one of the most significant immune components in teleost, IgM is the primary antibody involved in the innate immune response of fish [49]. Additionally, antioxidant enzymes including SOD, CAT, POD, GSH-Px, and GR are essential constituents of the complex immune system and key components of the enzymatic defense mechanism protecting organisms from oxidative damage [20, 21]. MDA is the end product of lipid peroxidation and an important indicator of oxidative stress, which can be used to evaluate free radical activity [21]. The biological production of NO plays a key role in nonspecific host defense; but NO overproduction can also damage animal tissue health by stimulating tumorigenesis [50]. In this study, low inclusion level (≤17.2%) of CSM generally enhanced the plasma IgM level and hepatic GR activity, while higher CSM inclusion (≥25.7%) generally increased the plasma MDA level but decreased the plasma AKP activity as well as hepatic SOD and GR activities. These findings indicated that lower CSM concentrations enhanced the immune capacity of *H. wyckioides* to some extent, but higher dietary CSM inclusion may depress the antioxidant capacity of *H. wyckioides*, which may be mainly related to the free gossypol present in CSM. The suppression of free gossypol on antioxidant capability has been verified in other aquatic animals including Ussuri catfish [5], grass carp [7], and Pacific white shrimp *Litopenaeus vannamei* [51].

Previous studies have shown that dietary FM replacement with CSM has different effects on the proximate compositions of different fish species. The whole-body protein [8] or lipid [9, 52] contents of fish showed a decreasing trend with the increasing replacing levels of FM by CSM, while dietary CSM inclusion has no significant effect on the proximate composition of blunt snout bream [6] and grass carp [7]. In this study, the whole-body crude lipid content of *H. wyckioides* showed a decreasing trend with the increased dietary CSM inclusion level, which might be mainly related to the free gossypol present in CSM. The potential toxicological effects of dietary free gossypol have been shown to impair the protein and lipid deposition of fish [9, 32, 52, 53]. For example, tilapia fed CSM-based diet had a lower whole-body lipid level than fish fed CSM-based diet supplemented with iron for detoxification of gossypol [53]; the whole-body lipid content of channel catfish linearly decreased with the increasing dietary gossypol concentrations [32].

## 5. Conclusion

The suitable CSM inclusion (17.2%) enhanced the amino acids balance (increased arginine content), then improved the digestive enzymes activities and protein metabolism, and thereby promoted the growth performance of *H. wyckioides*. However, excess dietary CSM inclusions (≥34.4%) overall decreased the digestive enzyme activity and antioxidant capacity of *H. wyckioides*, and thereby depressed the growth rate of *H. wyckioides*, which may be attributed to excess free gossypol in high CSM-based diets. Additionally, appropriate amount of CSM (17.2%) inclusion in diets for *H. wyckioide* was helpful to reduce the feed cost. CSM is a potentially cost-effective alternative plant protein source in aquafeeds.

## Figures and Tables

**Table 1:** Ingredients and proximate composition (% dry matter) of five experimental diets.

	Price^6^	Dietary inclusion levels of cottonseed meal				
	US$/kg	0%	8.5%	17.2%	25.7%	34.4%
*Ingredients*						
Fish meal^1^	1.90	52.00	45.80	39.50	33.30	27.00
Cottonseed meal^1^	0.89	0.00	8.50	17.20	25.70	34.40
Fish oil	2.41	0.90	1.40	1.90	2.40	2.90
Soybean oil	1.47	4.80	4.75	4.70	4.65	4.60
DL-Methionine^3^	2.93	0.00	0.09	0.19	0.28	0.38
L-Lysine^3^	1.36	0.00	0.17	0.34	0.51	0.68
L-leucine	9.14	0.00	0.08	0.16	0.25	0.33
Wheat middling	0.49	25.55	24.46	23.26	21.16	19.96
*α*-Starch	0.93	13.00	11.00	9.00	8.00	6.00
Soybean lecithin	0.96	0.50	0.50	0.50	0.50	0.50
Vitamin C^2^	2.29	0.02	0.02	0.02	0.02	0.02
Ca(H_2_PO_4_)_2_	0.61	1.20	1.20	1.20	1.20	1.20
NaCl	0.09	0.20	0.20	0.20	0.20	0.20
Ethoxyquin (30%)	4.57	0.03	0.03	0.03	0.03	0.03
Choline chloride (40%)	1.04	0.30	0.30	0.30	0.30	0.30
Vitamin mixture^4^	4.29	0.50	0.50	0.50	0.50	0.50
Mineral mixture^5^	1.86	0.50	0.50	0.50	0.50	0.50
*Proximate composition*						
Dry matter		90.79	88.09	89.85	89.04	88.61
Crude protein		40.72	41.41	41.45	41.27	41.12
Crude lipid		10.88	10.43	10.06	10.69	10.62
Ash		11.06	10.80	10.31	9.74	9.28
Cross energy (KJ/g)		20.59	20.40	20.61	20.71	20.72
Free gossypol (mg/g)		−	0.10	0.20	0.29	0.39
Feed price (US$/kg)		1.40	1.35	1.31	1.27	1.23

^1^Supplied by Kunming Tianyuan Feed Co., Ltd. (Yunnan, China); fish meal, 73.6% crude protein, 9.0% crude lipid; cottonseed meal, 49.6% crude protein, 1.3% crude lipid; wheat middling, 13.5% crude protein, 0.86% crude lipid. ^2^L-Ascorbate-2-polyphosphate (35%), supplied by Galaxy Chemicals Co., Ltd. (Hubei, China). ^3^Supplied by Shanghai Hanhong Chemical Co., Ltd. (Shanghai, China). ^4^Vitamin premix (g/kg of mixture): retinyl acetate (2800000 IU/g), 2; cholecalciferol, 0.03; DL-*α*-tocopheryl acetate, 30; menadione, 3; thiamine hydrochloride, 8; riboflavin, 11; pyridoxine hydrochloride, 8; vitamin B_12_, 0.02; ascorbic acid, 50; folic acid, 1; biotin, 0.1; niacin, 30; calcium D-pantothenate, 32; and inositol, 25. ^5^Mineral premix (g/kg of mixture): MgSO_4_•7H_2_O, 180; KI, 1; FeSO_4_•H_2_O, 260; ZnSO_4_•H_2_O, 180; CuSO_4_• 5H_2_O, 25; Na_2_Se_2_O_3_, 0.01; MnSO_4_•H_2_O, 180; and CoCl_2_•6H_2_O, 0.75. ^6^Price of feed ingredients is calculated with the exchange rate of US$ to RMB at 7.00.

**Table 2:** Amino acid composition of the experiment diets (% dry matter).

	Dietary inclusion levels of cottonseed meal				
	0%	8.5%	17.2%	25.7%	34.4%
*Essential amino acids*					
Arginine	2.16	2.42	2.69	2.94	3.21
Histidine	1.36	1.32	1.27	1.23	1.18
Isoleucine	1.40	1.35	1.30	1.24	1.19
Leucine	2.54	2.55	2.56	2.54	2.57
Lysine	2.86	2.89	2.87	2.83	2.86
Methionine	1.01	1.04	1.02	1.05	1.03
Phenylalanine	1.52	1.55	1.59	1.62	1.66
Threonine	1.40	1.35	1.30	1.25	1.20
Valine	1.96	1.92	1.88	1.83	1.79
Tryptophan^1^	0.44	0.43	0.43	0.42	0.41
*Non-essential amino acids*					
Alanine	2.15	2.04	1.92	1.80	1.69
Aspartic acid	3.40	3.38	3.36	3.33	3.31
Cysteine	0.36	0.37	0.38	0.39	0.39
Glutamic acid	6.42	6.64	6.87	7.03	7.26
Glycine	2.39	2.28	2.17	2.06	1.95
Proline	1.84	1.80	1.76	1.70	1.66
Serine	1.37	1.37	1.38	1.37	1.38
Tyrosine	1.06	1.04	1.02	0.99	0.97

^1^Tryptophan content of experimental diets was estimated according to the ingredient amino acid composition. The content of tryptophan in fish meal, cottonseed meal, and wheat middling was 0.76, 0.51, and 0.17% of dry matter, respectively.

**Table 3:** Sequence of primers used for real-time quantitative PCR.

Name	Primer sequence (5′−3′)		Length (bp)	Tm (°C)
AMPD1	Forward	CCACTATTGACCCACAGTCATACC	24	56.7
	Reverse	TATGCTTGGATTCATCGTCAACAC	24	
GDH	Forward	TCAAAATCAACCCCAAAAACTTCT	24	59.6
	Reverse	ATCAGGGGCAGGGACATCAATA	22	
IGF-1	Forward	GGGGACCGGGGCTTTTATT	19	60.1
	Reverse	GTGTGCCGTTGCTCTCGTA	19	
mTOR	Forward	AAGCCGCGTCACATCACACC	20	59.1
	Reverse	ATCAAAGCGCTCCTCCATCAG	21	
*β*-Actin	Forward	GGCCGTGACCTGACTGAATACCTC	24	61.3
	Reverse	AATGCCCATCTCCTGCTCAAAGTC	24	

AMPD1: adenosine monophosphate deaminase 1; GDH: glutamate dehydrogenase; IGF-1: insulin-like growth factors-1; mTOR: mammalian target of rapamycin.

**Table 4:** Growth performance of *Hemibagrus wyckioides* fed diets with various level of cottonseed meal.

	Dietary inclusion levels of cottonseed meal					Regression analysis		
	0%	8.5%	17.2%	25.7%	34.4%	Regression equation	*R* ^2^	*P* value
Final body weight (g)	20.64 ± 0.62^ab^	20.25 ± 0.77^ab^	23.23 ± 0.88^b^	20.74 ± 1.24^ab^	19.90 ± 1.03^a^	*y* = 20.240 + 14.352*x*–31.630*x*^2^	0.192	0.278
Weight gain	5.50 ± 0.22^ab^	5.32 ± 0.18^ab^	6.32 ± 0.26^b^	5.57 ± 0.41^ab^	5.29 ± 0.34^a^	*y* = 5.353 + 4.512*x*–9.673*x*^2^	0.174	0.318
DGC (%/day)	2.27 ± 0.05^ab^	2.23 ± 0.05^ab^	2.47 ± 0.06^b^	2.28 ± 0.10^ab^	2.21 ± 0.09^a^	*y* = 2.240 + 1.092*x*–2.397*x*^2^	0.176	0.313
Feed intake (g/kg MBW/d)	8.21 ± 0.22	8.21 ± 0.14	8.81 ± 0.21	8.57 ± 0.30	8.66 ± 0.33	*y* = 8.244 + 1.039*x*	0.172	0.124
FCR	0.89 ± 0.01^a^	0.90 ± 0.01^a^	0.90 ± 0.01^a^	0.93 ± 0.02^ab^	0.96 ± 0.02^b^	*y* = 0.885 + 0.133*x*	0.459	0.006
PER	2.75 ± 0.03^c^	2.66 ± 0.04^abc^	2.69 ± 0.02^bc^	2.61 ± 0.05^ab^	2.53 ± 0.06^a^	*y* = 2.747–0.414*x*	0.543	0.002
Feed cost (US$/kg fish gain)	1.25 ± 0.01^b^	1.22 ± 0.02^ab^	1.17 ± 0.01^a^	1.18 ± 0.02^ab^	1.18 ± 0.01^ab^	*y* = 1.251–0.382*x* + 0.493*x*^2^	0.588	0.005
Survival rate (%)	98.89 ± 1.11	96.67 ± 1.93	98.89 ± 1.11	96.67 ± 1.93	96.67 ± 1.93	*y* = 98.445–3.706*x*	0.060	0.380

Values are presented as means ± standard error (*n* = 3). Mean values in the same row with different superscripts are significant different (*P* < 0.05). DGC: daily growth coefficient; MBW: mean metabolic body weight; FCR: feed conversion rate; PER: protein efficiency ratio; feed cost is converted from RMB into US$ with the exchange rate of US$ to RMB at 7.00.

**Table 5:** Digestive enzymes activities in the gastrointestinal tract of *Hemibagrus wyckioides* fed diets with various level of cottonseed meal.

	Dietary inclusion levels of cottonseed meal					Regression analysis		
	0%	8.5%	17.2%	25.7%	34.4%	Regression equation	*R* ^2^	*P* value
*Stomach*								
Pepsin (U/mg protein)	15.67 ± 0.35^a^	16.33 ± 1.90^a^	20.25 ± 1.22^b^	15.67 ± 0.85^a^	15.46 ± 0.39^a^	*y* = 15.430 + 23.471*x*–50.761*x*^2^	0.266	0.157
*Intestine*								
Trypsin (U/*μ*g protein)	1.38 ± 0.06^b^	1.14 ± 0.03^ab^	1.19 ± 0.12^ab^	0.98 ± 0.11^a^	1.04 ± 0.02^a^	*y* = 1.309–0.683*x*	0.445	0.007
Lipase (U/g protein)	39.46 ± 3.93	38.00 ± 3.78	33.40 ± 2.13	46.29 ± 6.99	36.68 ± 3.17	*y* = 38.217 + 2.292*x*	0.003	0.853
Amylase (U/mg protein)	2.70 ± 0.27^ab^	3.07 ± 0.29^ab^	3.25 ± 0.07^b^	2.32 ± 0.22^a^	2.63 ± 0.27^ab^	*y* = 2.792 + 2.198*x*–6.101*x*^2^	0.167	0.334

Values are presented as means ± standard error (*n* =3). Mean values in the same row with different superscripts are significant different (*P* <0.05).

**Table 6:** Protein metabolism-related parameters in plasma and liver of *Hemibagrus wyckioides* fed diets with various level of cottonseed meal.

	Dietary inclusion levels of cottonseed meal					Regression analysis		
	0%	8.5%	17.2%	25.7%	34.4%	Regression equation	*R* ^2^	*P* value
*Plasma*								
Albumin (g/L)	15.34 ± 2.19	16.09 ± 1.08	13.47 ± 1.50	11.97 ± 0.99	12.72 ± 1.14	*y* = 15.791–7.797*x*	0.264	0.050
BUN (mmol/L)	9.86 ± 1.01	10.96 ± 1.26	13.56 ± 1.54	10.27 ± 0.63	12.51 ± 1.07	*y* = 10.511 + 3.844*x*	0.093	0.268
Insulin (mU/L)	8.11 ± 0.45	8.25 ± 1.20	7.80 ± 0.40	8.52 ± 0.81	8.35 ± 0.59	*y* = 8.057 + 0.628*x*	0.010	0.728
GH (*μ*g/L)	17.60 ± 0.31^a^	23.61 ± 0.43^c^	24.81 ± 1.30^c^	25.09 ± 0.83^c^	21.10 ± 0.56^b^	*y* = 17.758 + 56.866*x*–103.737*x*^2^	0.851	<0.001
IGF-1 (*μ*g/L)	19.90 ± 1.28	22.33 ± 0.23	21.08 ± 2.49	20.19 ± 1.14	18.89 ± 0.92	*y* = 20.294 + 13.438*x*–35.202*x*^2^	0.199	0.264
GDH (U/L)	9.63 ± 0.42^b^	6.04 ± 0.43^a^	5.95 ± 0.61^a^	7.06 ± 0.33^a^	6.38 ± 0.50^a^	*y* = 9.115–21.297*x* + 34.838*x*^2^	0.568	0.007
AMPD (U/L)	14.05 ± 0.23^a^	19.53 ± 0.98^ab^	24.39 ± 0.32^b^	20.25 ± 2.84^ab^	20.39 ± 3.25^ab^	*y* = 14.234 + 57.976*x*–97.520*x*^2^	0.484	0.019
AST (U/L)	29.21 ± 0.19^c^	22.77 ± 1.67^c^	20.89 ± 1.46^b^	12.47 ± 0.80^b^	7.82 ± 0.73^a^	*y* = 29.249–44.236*x*	0.935	<0.001
ALT (U/L)	12.26 ± 0.78	13.93 ± 0.62	10.10 ± 2.14	10.77 ± 0.69	10.11 ± 1.02	*y* = 12.925–6.214*x*	0.222	0.076
*Liver*								
GDH (U/g protein)	0.17 ± 0.00	0.15 ± 0.01	0.15 ± 0.01	0.16 ± 0.01	0.15 ± 0.01	*y* = 0.161–0.019*x*	0.077	0.318
AMPD (U/g protein)	0.71 ± 0.02^a^	4.15 ± 0.20^b^	0.99 ± 0.07^a^	0.74 ± 0.06^a^	0.73 ± 0.03^a^	y = 1.565 + 6.682*x*–19.742*x*^2^	0.246	0.183
*γ*-GT (U/g protein)	0.17 ± 0.01^a^	0.39 ± 0.04^b^	0.36 ± 0.04^b^	0.36 ± 0.04^b^	0.42 ± 0.03^b^	y = 0.202 + 1.093*x*–1.438*x*^2^	0.571	0.006
AST (U/g protein)	11.00 ± 1.20^a^	28.61 ± 1.12^b^	27.82 ± 1.89^b^	22.49 ± 3.87^b^	21.75 ± 1.49^b^	y = 13.362 + 111.039*x*–204.613*x*^2^	0.592	0.005
ALT (U/g protein)	12.46 ± 2.26	16.62 ± 2.18	15.07 ± 1.84	18.65 ± 3.66	14.41 ± 2.26	y = 12.586 + 32.723*x*–57.854*x*^2^	0.155	0.363

Values are presented as means ± standard error (*n* = 3). Mean values in the same row with different superscripts are significant different (*P* < 0.05). BUN: blood urea nitrogen; GH: growth hormone; IGF-1: insulin-like growth factors-1; GDH: glutamate dehydrogenase; AMPD: adenosine monophosphate deaminase; AST: aspartate aminotransferase; ALT: alanine aminotransferase; *γ*-GT: *γ*-glutamyl transferase.

**Table 7:** Relative mRNA expression of protein metabolism-related enzymes in *Hemibagrus wyckioides* fed diets with various level of cottonseed meal.

	Dietary inclusion levels of cottonseed meal					Regression analysis		
	0%	8.5%	17.2%	25.7%	34.4%	Regression equation	*R* ^2^	*P* value
*Liver*								
IGF-1	1.04 ± 0.29^b^	0.46 ± 0.11^a^	0.14 ± 0.01^a^	0.63 ± 0.07^ab^	0.52 ± 0.04^ab^	*y* = 0.981–4.908*x* + 8.705*x*^2^	0.559	0.057
mTOR	1.01 ± 0.10^a^	3.92 ± 0.48^b^	2.73 ± 0.83^ab^	0.93 ± 0.04^a^	1.49 ± 0.05^a^	*y* = 1.813 + 11.421*x*–28.286*x*^2^	0.310	0.188
AMPD1	1.01 ± 0.12^b^	0.05 ± 0.02^a^	0.08 ± 0.03^a^	0.02 ± 0.01^a^	0.05 ± 0.03^a^	*y* = 0.856–5.769*x* + 8.799*x*^2^	0.792	<0.001
GDH	1.00 ± 0.01^b^	0.42 ± 0.03^a^	0.73 ± 0.06^b^	0.39 ± 0.12^a^	0.76 ± 0.16^b^	*y* = 0.912–3.167*x* + 5.761*x*^2^	0.344	0.121
*Muscle*								
AMPD	1.02 ± 0.20^ab^	0.41 ± 0.03^a^	1.43 ± 0.42^b^	0.76 ± 0.02^ab^	1.56 ± 0.07^b^	*y* = 0.749 + 1.187*x*	0.181	0.220
GDH	1.00 ± 0.01^b^	0.41 ± 0.01^a^	0.40 ± 0.02^a^	0.29 ± 0.06^a^	0.25 ± 0.01^a^	*y* = 0.933–3.645*x* + 4.828*x*^2^	0.872	<0.001

Values are presented as means ± standard error (*n* = 3). Mean values in the same row with different superscripts are significant different (*P* < 0.05). IGF-1: insulin-like growth factors-1; mTOR: mammalian target of rapamycin; AMPD1, adenosine monophosphate deaminase 1; GDH, glutamate dehydrogenase.

**Table 8:** Antioxidant-related parameters in plasma and liver of *Hemibagrus wyckioides* fed diets with various level of cottonseed meal.

	Dietary inclusion levels of cottonseed meal					Regression analysis		
	0%	8.5%	17.2%	25.7%	34.4%	Regression equation	*R* ^2^	*P* value
*Plasma*								
IgM (g/L)	0.05 ± 0.01^a^	0.15 ± 0.01^c^	0.15 ± 0.03^c^	0.13 ± 0.01^bc^	0.09 ± 0.01^ab^	*y* = 0.061 + 0.743*x*–1.455*x*^2^	0.657	0.002
MDA (nmol/ml)	7.21 ± 0.82^a^	7.52 ± 0.43^a^	8.37 ± 1.05^a^	11.40 ± 0.14^a^	22.56 ± 2.55^b^	*y* = 4.497 + 28.814*x*	0.652	<0.001
AKP (U/dl)	15.18 ± 2.41^b^	17.66 ± 2.56^b^	6.38 ± 1.19^a^	2.74 ± 0.72^a^	3.49 ± 0.74^a^	*y* = 16.747–31.908*x*	0.669	<0.001
*Liver*								
SOD (U/mg protein)	34.46 ± 0.73^b^	32.07 ± 2.01^ab^	29.94 ± 1.78^ab^	33.65 ± 2.95^ab^	27.72 ± 1.30^a^	*y* = 33.946–9.580*x*	0.231	0.047
CAT (U/mg protein)	10.46 ± 0.30	10.81 ± 0.35	10.97 ± 0.20	10.16 ± 0.51	10.07 ± 0.33	*y* = 10.531 + 2.677*x*–7.365*x*2	0.303	0.115
POD (U/mg protein)	1.17 ± 0.14	1.08 ± 0.08	1.13 ± 0.05	1.08 ± 0.03	0.97 ± 0.03	*y* = 1.167–0.328*x*	0.216	0.081
GSH-Px (U/*μ*g protein)	0.14 ± 0.00	0.11 ± 0.01	0.11 ± 0.01	0.12 ± 0.01	0.12 ± 0.01	*y* = 126.150–23.065*x*	0.115	0.216
GR (U/g protein)	7.99 ± 0.85^ab^	9.21 ± 1.09^b^	12.78 ± 1.28^c^	6.38 ± 0.12^ab^	5.50 ± 0.34^a^	*y* = 8.452 + 14.149*x*–37.782*x*^2^	0.351	0.049
AKP (U/g protein)	12.20 ± 1.57	15.77 ± 2.86	14.86 ± 2.93	10.93 ± 1.06	11.19 ± 1.55	*y* = 14.262–5.136*x*	0.068	0.347
LDH (U/mg protein)	4.07 ± 0.12	4.41 ± 0.24	3.93 ± 0.31	4.39 ± 0.19	3.64 ± 0.38	*y* = 4.088 + 1.969*x*–5.142*x*2	0.219	0.227
NO (*μ*mol/g protein)	6.64 ± 0.40^b^	4.54 ± 0.42^a^	4.71 ± 0.81^ab^	5.85 ± 0.42^ab^	5.25 ± 0.71^ab^	*y* = 6.080–6.609*x* + 10.073*x*^2^	0.124	0.453

Values are presented as means ± standard error (*n* = 3). Mean values in the same row with different superscripts are significant different (*P* < 0.05). IgM: immunoglobulin M; MDA: malondialdehyde; AKP: alkaline phosphatase; SOD: superoxide dismutase; CAT: catalase; POD: peroxidase; GSH-Px: glutathione peroxidase; GR: glutathione reductase; LDH: lactate dehydrogenase; NO: nitric oxide.

**Table 9:** The whole-body composition of *Hemibagrus wyckioides* fed diets with various level of cottonseed meal.

	Initial	Dietary inclusion levels of cottonseed meal					Regression analysis		
		0%	8.5%	17.2%	25.7%	34.4%	Regression equation	*R* ^2^	*P* value
Moisture (%)	85.47	69.76 ± 0.20^a^	70.77 ± 0.11^ab^	70.28 ± 0.44^ab^	70.47 ± 0.49^ab^	71.26 ± 0.32^b^	*y* = 69.971 + 2.244*x*	0.300	0.035
Crude protein (%)	11.58	14.90 ± 0.09	15.13 ± 0.04	14.90 ± 0.06	15.22 ± 0.33	14.88 ± 0.20	*y* = 14.917 + 1.393*x*–2.827*x*^2^	0.056	0.707
Crude lipid (%)	1.47	10.75 ± 0.06^b^	10.26 ± 0.14^ab^	10.51 ± 0.07^b^	10.09 ± 0.35^ab^	9.57 ± 0.33^a^	*y* = 10.746–2.125*x*	0.491	0.004
Ash (%)	1.72	3.02 ± 0.03	3.11 ± 0.06	3.07 ± 0.06	3.04 ± 0.11	3.02 ± 0.01	*y* = 3.036 + 0.452*x*–1.075*x*^2^	0.087	0.580

Values are presented as means ± standard error (*n* = 3). Mean values in the same row with different superscripts are significant different (*P* < 0.05).

## Data Availability

Data supporting the results in the present study are available from the corresponding author upon legitimate request.
